# Prevalence of Iron Deficiency, Anemia, and Associated Factors in a Blood Donor Population in Brazzaville, Congo

**DOI:** 10.1155/2023/8827984

**Published:** 2023-12-13

**Authors:** Firmine Olivia Galiba Atipo-Tsiba, Earl Quincy Gayaba Mouyabi, Brunel Monic Angounda, Serge Oscar Mokono, Lethso Thibaut Ocko Gokaba, Alexis Elira Dokekias

**Affiliations:** ^1^Hematology Department, Teaching Hospital of Brazzaville, Brazzaville, Congo; ^2^Faculty of Health Sciences, Marien Ngouabi University, Brazzaville, Congo; ^3^National Center for Blood Transfusion, Brazzaville, Congo; ^4^Laboratory of Hematology, Teaching Hospital of Brazzaville, Congo

## Abstract

**Introduction:**

Blood donation is not without risk to the donor. It results in a substantial loss of iron and decreased hemoglobin. In our country, no predonation assessment is carried out and the selection of blood donors is only clinical.

**Objectives:**

To determine the prevalence of iron deficiency, anemia, and iron deficiency anemia and to identify the factors associated with anemia and iron status in a blood donor population at the National Center for Blood Transfusion (NCBT). *Methodology*. A prospective study is carried out that consists of 120 blood donors in three NCBT branches in the capital from June to November 2021. The donors were divided into 3 groups: first time donors (FTDs), occasional donors (ODs) who have already made between 1 and 3 previous donations, and regular donors (RDs) with at least 4 previous donations. Iron deficiency was defined by a serum ferritin value of less than 30 ng/mL in men and 20 ng/mL in women. Anemia was defined by Hb levels below 13 g/dL in men and 12 g/dL in women. Iron deficiency anemia was defined by association of anemia and iron deficiency. The chi-square test was used for the comparison of the proportions. The odds ratio with the 95% confidence interval was calculated to assess the association between two variables. The *p* value of the probability was considered significant for a value < 0.05.

**Results:**

Mean serum ferritin and hemoglobin values were lower in RD in both sexes. The prevalence of iron deficiency, anemia, and iron deficiency anemia were 16.66%, 31.66%, and 10.83%, respectively. The factors associated with the three abnormalities were female sex, donor type, including RD, and number of previous donations.

**Conclusion:**

Iron deficiency, anemia, and iron deficiency anemia are common among blood donors in Brazzaville. Anemia affects almost a third of blood donors and is not always linked to iron deficiency. Safety of donors should be improved by systematic measurement of ferritinemia and hemoglobin levels before allowing donations for appropriate management in the event of abnormalities.

## 1. Introduction

Blood transfusion is an essential replacement therapy for many patients during various medical and surgical conditions. Transfusions of erythrocyte concentrates are performed in dozens as part of intensive oncology treatment, chronic anemia, a severe hemorrhagic accident, or an organ transplant. Blood donation is regulated by regulations whose purpose is, on the one hand, to guarantee the safety of blood transfusions made by blood products derived from these donations and, on the other hand, to preserve the health of donors. To reduce the infectious risk of transfusion, WHO recommends the use of voluntary but regular blood donors. Thus, they constitute a population at risk of iron deficiency and of anemia [1, 2]. Blood donation results in a substantial loss of iron and a decrease of approximately 1 g/dL in hemoglobin (Hb) with each collection procedure, during which up to 425 to 475 ml of total blood is collected [3, 4].

To prevent these risks and avoid inappropriate donations, a number of strategies are being implemented by some teams, including limiting the annual number of blood donations and predonation screening for iron deficiency and anemia leading to deferral of blood donation below a certain threshold of eligibility for blood donation [2, 5, 6]. In Africa, its practice is not systematic, and several studies have shown that iron deficiency and anemia in general are frequent and can affect, respectively, up to 63% and 36.5% of blood donors [7–11]. Furthermore, iron deficiency was the main but not exclusive cause of anemia. In the Republic of Congo, the practice of blood transfusion is important, and every year, more than 40,000 blood donations are made. These are preceded by a medical examination, including an interview aimed at screening for conditions that are contraindicative of donation and assessment of general health [12]. However, no hemoglobin and ferritinemia estimation is performed. Thus, in order to strengthen the safety of blood donors, we carried out this study, the first of its kind, whose objective was to determine the frequency of iron deficiency, anemia, and iron deficiency anemia in a population of blood donors and identify the associated factors.

## 2. Methodology

It was an analytical cross-sectional study that took place over a six-month period from June 1 to November 30, 2021. It was multicentric and was carried out in three of the four stations responsible for blood collection in Brazzaville, capital of the Republic of Congo: those of the teaching hospital, the specialized hospital Mère-Enfants Blanche Gomes, and the reference hospital of Makélékélé. Blood donors were recruited consecutively during the predonation interview. Those who met the eligibility criteria for blood donation, being between the ages of 18 and 60 years old, weighing 55 kg or more, and qualified by medical selection were selected for the study, having given their consent to participate. Those whose sampling process has not been completed due to difficulty in drawing blood and those whose determination of ferritinemia could not be carried out for hemolysed serum were excluded. The variables analyzed were sociodemographic (age, sex, type of donor, and number of previous donations) and biological (Hb level and ferritinemia). Three types of blood donors have been identified, all unpaid:

- *First-time donors (FTD)*. Donors who never had donated blood in the past
- *Occasional donors (OD)*. Donors who have previously made between 1 and 3 donations in their lifetime regardless of the time between donations and/or since the last donation
- *Regular donors (RD)*. Donors who have previously made at least 4 donations in their lifetime regardless of the time between donations and/or since the last donation

Before the total blood donation, 10 ml of blood was collected from a crease elbow vein: 5 ml on an EDTA tube for hemoglobin determination and 5 ml on a dry tube for ferritinemia. The analyses were carried out at the Faculty's Laboratory of Training, Research, and Analysis of Medical Biology of Health Sciences at Marien Ngouabi University in Brazzaville. The blood count was carried out on the HORIBA MEDICAL Yumizen H550 machine. The determination of ferritinemia was carried out using the Bio Mérieux mini vidas semiautomaton using the ELFA technique. Iron deficiency was defined by a ferritinemia value of less than 30 ng/mL in men and 20 ng/mL in women. Anemia was defined by a Hb level of less than 13 g/dL in men and 12 g/dL in women. Iron deficiency anemia was defined by association of anemia and iron deficiency.

The influence of the dependent variables was highlighted, thanks to the application of Fisher's exact test on our data. The statistical difference observed between the qualitative variables was assessed by the Pearson Khi2 test. The *p* value of the probability was considered significant for a value < 0.05.

## 3. Results

Table 1 shows the distribution of a population of blood donors by sociodemographic characteristics in Brazzaville, Congo.

The sex ratio was 4/1.

The average age was 34 years ± 10 years with extremes of 18 and 60 years.

Among the 26 women, there were 24 (92.31%) FTD, 02 (07.69%) RD, and no OD. Among the 94 men, there were 59 (62.77%) FTD, 27 (28.72%) RD, and 8 (8.51%) OD. The median value of ferritinemia in blood donors was 74.03 ± 16.74 ng/mL. It was 88.46 ± 14.76 ng/mL ± in men and 53.08 ± 22.94 ng/mL ± in women. The median Hb value was 13.80 ± 2.81 g/dL. It was 14.25 ± 1.94 g/dL in men and 11.70 ± 4.58 g/dL in women.

Table 2 presents the mean values of ferritinemia and Hb levels in a blood donor population by the blood donor type and sex in Brazzaville.

Table 3 shows the prevalence of iron deficiency, anemia, and iron deficiency anemia in a blood donor population in Brazzaville, Congo.

Among the 20 blood donors with iron deficiency, 13 or 65% were in the anemia stage and the remaining 7 were in the preanemic stage.

Concerning the 38 blood donors who had anemia, it was linked to iron deficiency in 13 cases (34.21% of anemia). Anemia was therefore undetermined in 65.79%.

Table 4 shows the correlation between the presence of iron deficiency, anemia, and iron deficiency anemia in blood donors and the sex, type of donor, and number of previous blood donations.

## 4. Discussion

The male predominance of blood donors (78.33% of cases) observed in our study is common in sub-Saharan Africa, as shown by several studies: 79.9%, 80.6%, and 89.3%, respectively, in Nigeria [13], DRC [8], and Ghana [10], up to 95.1% in our country [12]. These high proportions could be explained among other things by the contraindications of blood donation that are numerous in women, especially breastfeeding, menstruation, or pregnancy less than 6 months old. However, cultural causes should be sought, since studies carried out in Western countries report almost equal proportions of male and female blood donors [6, 14] more globally; WHO reports that 33% of blood donations come from women [1].

The majority of blood donors in our country remain replacement donors as reported in a previous 10-year study [12]. With a shortage of blood products in the country, families often have no choice but to donate replacement blood and hope to benefit from a blood product in the future. According to WHO data, of the 118.5 million blood donations collected annually worldwide, 40% are collected in high-income countries, where 16% of the world's population lives. There are 31.5 blood donations per 1,000 population in high-income countries, compared to 5.0 in low-income countries, which still collect more than 50% of their blood supply through offsetting or paid donations [1].

The design limitation in our study is the fact that the subclinical inflammation using at least CRP was not screened, knowing that ferritin is an acute phase protein and will be artificially increased by inflammation. This is apparent in the results since ferritin levels were as high as 1200 ng/mL in some participants which is indicative of either inflammation, and/or iron overload. It also implies that the prevalence estimated for iron deficiency or iron deficiency anemia might not reflect the true picture in the study population since some iron-deficient individuals might have been qualified as not being iron-deficient.

Iron deficiency in blood donors is a global problem [3, 8, 14–19]. The frequency found in our series, of 16.66%, seems low compared to those reported in Africa: 17.5% in a large population of nearly 4,500 blood donors in South Africa [15], 20.6% in Nigeria [16], just over a quarter (27.4%) in Ghana [10], 35.2% in Algeria [9], or 63% in the DRC [8] to name but a few. But, as suggested above, the hemoglobin estimation might have been adversely affected by the absence of subclinical inflammation screening; therefore, anemia prevalence might have been underestimated. In the last two studies, the high proportions of iron deficiency could be explained by the fact that regular blood donors were the most numerous and the prevalence of iron deficiency was higher among these regular donors than among other types of donors. In lesser developed countries where iron deficiency is already a public health problem, the prevention of anemia in general and iron deficiency anemia, in particular, must be an essential issue for the safety of the blood donor but also for quantitative and qualitative self-sufficiency in blood products. Indeed, iron deficiency can be the cause of anemia with multiple clinical implications in relation to the decrease in oxygen transport in the body.

This prevention is based, on the one hand, on the postponement of donors whose Hb level is below a regulatory threshold and, on the other hand, on the prevention of iron deficiency. Several measures can be used: the increase in the minimum interval between two total blood donations and/or the reduction in the maximum annual frequency of donations; the assay of serum ferritin measuring iron reserves and finally donor iron supplementation [2, 17, 18, 20–22]. In France, after modeling different scenarios measuring the loss of donations, it was decided to opt for a ferritinemia strategy directed at groups at risk of iron deficiency with postponement of 6 months if ferritinemia <15 ng/ml and spacing of donations if between 15 and 25 ng/ml [17]. Regular monitoring of ferritinemia allows individualization of the blood donation calendar in Canada [23]. In the face of the ongoing challenge of the blood supply facing many African countries [1, 24], it is not easy to address the problem of iron deficiency and anemia in front-line blood donors and reach a consensus. Some experts oppose systematic substitution treatment after donation, fearing to mask an iron deficiency not related to the donation [17]. Determining suitability for donation through isolated dosing of Hb prior to donation would at least not aggravate preexisting anemia.

In our study, nearly, a third of donors had anemia (31.66%) of which 34.21% were iron-deprived, confirming some observations that iron deficiency accounts for a significant share of anemia in blood donors, especially the most regular [25–27]. More than 65% of the anemia is not being attributed to iron deficiency. This high proportion of anemia illustrates the need for a predonated biological assessment to diagnose, explore, and adequately manage potential future donors. Throughout the world, the most common cause of anemia is martial deficiency, due to prolonged deficiency resulting from inadequate dietary iron intake, increased requirements during growth or pregnancy, and increased losses due to menstruation or helminthiasis. In Africa, and more particularly in tropical zones, apart from genetic causes (especially sickle-cell anemia), other important causes include infections and nutritional deficiencies in folic acid and vitamin B12. In areas of high prevalency, anemia is an important complication of malaria [28, 29]. Blood donor management should be comprehensive, addressing both nutritional and non-nutritional causes of anemia as appropriate.

Factors associated with iron deficiency on the one hand, and with anemia and iron deficiency anemia on the other hand, were, respectively, the number of donations (especially after the third) and the female sex. These are factors traditionally reported in the literature [6, 7, 9, 17, 25, 30]. In the Nzengu-Lukusa study in the Democratic Republic of Congo, iron deficiency was associated with the male sex: 70.42% in men compared to 33.33% in women. This may be due to the fact that family donors were not included in the study and regular donors are primarily male [8].

## 5. Conclusion

Iron deficiency, anemia, and iron deficiency anemia are common among blood donors in Brazzaville, especially among women and RD. In addition, the risk increases with the number of donations. The safety of donors should be improved by simple measures such as systematic measurement of ferritinemia and measurement of Hb levels prior to allowing donations. This predonation screening of iron deficiency and anemia will make it possible to carry out an etiological survey and then a curative and preventive management adapted to the different causes.

## Figures and Tables

**Table 1:** Distribution of a population of blood donors by sociodemographic characteristics in Brazzaville, Congo.

Characteristics	*N* (120)	%
*Sex*		
Men	94	78.33
Women	26	21.67

*Age (years)*		
18–30	51	42.50
31–45	52	43.33
46–60	17	14.17

*Type of blood donor*		
RD^*∗*^	29	24.17
OD^*∗∗*^	08	06.67
FTD^*∗∗∗*^	83	69.16

^*∗*^Regular donors. ^*∗∗*^Occasional donors. ^*∗∗∗*^First time donors: most of them (76/83) were recruited as part of a replacement blood donation still known as a compensation donation, when a family member or relative has received a blood product.

**Table 2:** Mean values of ferritinemia and Hb levels in a blood donor population by the blood donor type and sex in Brazzaville, Congo.

	Men		Women	
	Mean	Max/Min	Mean	Max/Min
*Ferritinemia (ng/mL)*				
RD^*∗*^	64.82	1200.00/07.26	45.70	1200.00/09.52
OD^*∗∗*^	98.81	348.08/10.19	—	—
FTD^*∗∗∗*^	152.81	197.97/03.21	104.88	57.05/34.35

*Hb (g/dL)*				
RD^*∗*^	13.18	16.90/08.40	09.95	11.70/08.20
OD^*∗∗*^	14.40	15.60/10.10	—	—
FTD^*∗∗∗*^	14.60	17.80/09.20	12.66	25.00/06.60

^*∗*^Regular donors. ^*∗∗*^Occasional donors. ^*∗∗∗*^First time donors.

**Table 3:** Prevalence of iron deficiency, anemia, and iron deficiency anemia in a blood donor population in Brazzaville, Congo.

Parameters	*N* (120)	%
*Iron deficiency*		
Yes	**20**	**16.66**
No	100	83.34

*Anemia*		
Yes	**38**	**31.66**
No	82	68.34

*Iron deficiency anemia*		
Yes	**13**	**10.83**
No	107	89.17

**Table 4:** Correlation between the presence of iron deficiency, anemia, and iron deficiency anemia in blood donors and the sex, type of donor, and number of previous blood donations.

	Iron deficiency	OR	95% IC	*p*
*Sex*				
Male (*n* = 94)	13.83%			
Female (*n* = 26)	23.08%	1.8692	0.6322−5.5269	0.003
*Type of donor*				
RD^*∗*^ (*n* = 29)	27.58%	0.8750	0.1453−5.2702	0.04
OD^*∗∗*^ (*n* = 8)	25%			
FTD^*∗∗∗*^ (*n* = 83)	10.84%	0.3649	0.0638−2.0861	0.03
*Number of previous donations*				
0–3 (*n* = 93)	11.83%			
4 and more (*n* = 27)	29.63%	0.3186	0.1128−0.9000	0.02

	Anemia	OR	95% IC	*p*

*Sex*				
Male (*n* = 94)	25.53%	3.9773		
Female (*n* = 26)	57.69%		1.6078−9.8384	0.000
*Type of donor*				
RD^*∗*^ (*n* = 29)	44.82%	1^*∗∗*^2308	0^*∗∗*^2567−5^*∗∗*^8999	0.03
OD^*∗∗*^ (*n* = 8)	50%			
FTD (*n* = 83)	25.30%	0.3607	0.0830−1.5672	0.007
*Number of previous donations*				
1–3 (*n* = 93)	27.96%			
4 and more (*n* = 27)	48.15%	0.4179	0.1733−1.0079	0.04

	Iron deficiency anemia	OR	95% IC	*p*

*Sex*				
Male (*n* = 94)	7.44%			
Female (*n* = 26)	23.077%	3.7286	1.1299−12.303	0.002
*Type of donor*				
RD^*∗*^ (*n* = 29)	13.79%	2.0833	0.3063−14.1687	0.04
OD^*∗∗*^ (*n* = 8)	25%			
FTD^*∗∗∗*^ (*n* = 83)	8.43%	0.2763	0.0467−1.6348	0.07
*Number of previous donations*				
0–3 (*n* = 93)	9.68%			
4 and more (*n* = 27)	14.81%	0.6161	0.1739−2.1826	0.453794

^*∗*^Regular donors. ^*∗∗*^Occasional donors. ^*∗∗∗*^First time donors.

## Data Availability

The data used to support the findings of this study are included within the article.
